# Whole-genome sequencing identifies functional noncoding variation in *SEMA3C* that cosegregates with dyslexia in a multigenerational family

**DOI:** 10.1007/s00439-021-02289-w

**Published:** 2021-06-02

**Authors:** Amaia Carrion-Castillo, Sara B. Estruch, Ben Maassen, Barbara Franke, Clyde Francks, Simon E. Fisher

**Affiliations:** 1 Language and Genetics Department, Max Planck Institute for Psycholinguistics, Nijmegen, The Netherlands; 2 Basque Center on Cognition, Brain and Language, San Sebastian, Spain; 3 Centre for Language and Cognition Groningen, University of Groningen, Groningen, The Netherlands; 4 School of Behavioral and Cognitive Neurosciences, University Medical Centre Groningen, Groningen, The Netherlands; 5 Departments of Human Genetics and Psychiatry, Radboud University Medical Center, Nijmegen, The Netherlands; 6 Donders Institute for Brain, Cognition and Behaviour, Radboud University, Nijmegen, The Netherlands

## Abstract

Dyslexia is a common heritable developmental disorder involving impaired reading abilities. Its genetic underpinnings are thought to be complex and heterogeneous, involving common and rare genetic variation. Multigenerational families segregating apparent monogenic forms of language-related disorders can provide useful entrypoints into biological pathways. In the present study, we performed a genome-wide linkage scan in a three-generational family in which dyslexia affects 14 of its 30 members and seems to be transmitted with an autosomal dominant pattern of inheritance. We identified a locus on chromosome 7q21.11 which cosegregated with dyslexia status, with the exception of two cases of phenocopy (LOD = 2.83). Whole-genome sequencing of key individuals enabled the assessment of coding and noncoding variation in the family. Two rare single-nucleotide variants (rs144517871 and rs143835534) within the first intron of the *SEMA3C* gene cosegregated with the 7q21.11 risk haplotype. In silico characterization of these two variants predicted effects on gene regulation, which we functionally validated for rs144517871 in human cell lines using luciferase reporter assays. *SEMA3C* encodes a secreted protein that acts as a guidance cue in several processes, including cortical neuronal migration and cellular polarization. We hypothesize that these intronic variants could have a *cis*-regulatory effect on *SEMA3C* expression, making a contribution to dyslexia susceptibility in this family.

**Supplementary Information:**

The online version contains supplementary material available at 10.1007/s00439-021-02289-w.

## Introduction

Dyslexia is a prevalent human neurodevelopmental condition, characterized by a difficulty learning to read despite conventional instruction, adequate educational opportunities and IQ, and a lack of sensory impairments (Shaywitz et al. 1992). It shows familial clustering and has been reported to be moderately heritable in a range of samples, with heritability estimates from 0.3 to 0.8 (Peterson and Pennington 2015). Studies thus far indicate a complex multifactorial etiology involving genetic and environmental factors (Bishop 2015).

Multiple genetic variants are likely to act as risk factors contributing to the liability of dyslexia. Until recent years, much of the research on the molecular basis of dyslexia focused on a handful of candidate genes (e.g., *ROBO1, KIAA0319, DCDC2*, and *DYX1C1*) that were identified through linkage analysis in families, and then followed up via fine-mapping of association with common variants within those genes (see (Carrion-Castillo et al. 2013) for a review). In the last decade, genome-wide association scan (GWAS) studies have tried to identify other loci affecting reading ability by querying common genetic variants across the whole genome for association in a relatively unbiased manner (Luciano et al. 2013; Field et al. 2013; Gialluisi et al. 2014, 2019, 2020; Eicher et al. 2013; Truong et al. 2019; Price et al. 2020). These GWAS efforts have pointed to a number of promising loci. On the other hand, there are some unusual extended families with high frequency of dyslexia, in which it may follow a roughly Mendelian inheritance pattern (Fagerheim et al. 1999; Nopola-Hemmi et al. 2001; de Kovel et al. 2004). Studying these families in which one, or perhaps a few, genetic variants with substantial penetrances could contribute to dyslexia has already proven to be valuable for the identification of several dyslexia candidate genes. For example, a rare haplotype of *ROBO1* was found to cosegregate with dyslexia status in the majority of affected relatives of a large family, and the same gene was disrupted by a translocation in an unrelated case (Hannula-Jouppi et al. 2005); also, *DYX1C1* was identified as a potential candidate because of a chromosomal rearrangement that cosegregated with dyslexia in multiple members of one family (Taipale et al. 2003).

Next-generation sequencing (NGS) has revolutionized the genetic analysis of human diseases by enabling the systematic and rapid screening of common and rare mutations in the whole genome or whole exome. This has led to the discovery of several disease-causing genes underlying previously unsolved Mendelian diseases (Bamshad et al. 2011). Moreover, the technology has also been used to explore the extent to which rare alleles can explain the heritability of complex diseases and health-related traits. For instance, through family-based approaches for detecting de novo mutations, NGS has identified new genes involved in the genetic architecture of complex and heterogenic disorders such as autism spectrum disorder (Iossifov et al. 2014) and intellectual disability (Gilissen et al. 2014), and is beginning to shed new light on pathways underlying speech apraxia (Eising et al. 2019; Hildebrand et al. 2020; den Hoed and Fisher 2020). Unusual families with a high frequency of dyslexia provide an additional possibility to rank variants for potential causal links to a trait/disorder, according to how well they cosegregate with the phenotype and their likely functional consequences at the molecular level. For example, whole-exome sequencing (WES) in an extended Swedish family identified a two-base mutation (chr3:123264558-9, hg19), which resulted in an amino acid change (p.R229L) within the *CEP63* gene, partially cosegregating with dyslexia: seven out of ten of the affected individuals were carriers of the risk variant (Einarsdottir et al. 2015). Another study that reported ten putatively linked loci in a Finnish family used whole-exome and whole-genome sequencing (WGS) in two critical individuals of the family to identify a rare non-synonymous variant within the *NCAN* gene, on chromosome 19p13.11 (maximum NPL = 1.36) (Einarsdottir et al. 2017). The variant was present in seven out of eight affected individuals and one out of four unaffected family members (Einarsdottir et al. 2017).

In the current study, we took advantage of the multiplex family design and sequencing technologies to study a three-generation Dutch pedigree in which almost half of its 30 members have dyslexia. We searched for chromosomal regions that cosegregate with dyslexia within this family using linkage analysis based on a single-nucleotide polymorphism (SNP) genotype array and used WGS to characterize the genetic variants found in seven critical individuals at single-nucleotide and structural levels. Next, we used the haplotype scaffolding (from the genotype array) for imputation in the remainder of the family members, and focused on rare variation that was shared among individuals with dyslexia, with the aim of identifying likely highly penetrant variants. Finally, we assessed the putative biological role of the identified variants of interest with functional experiments in human cell lines. This strategy can lead to the identification of new genetic variants involved in dyslexia, and promises to yield new clues on genes and pathways that are important for the development of reading abilities.

## Materials and methods

### Sample

**Fig. 1:**
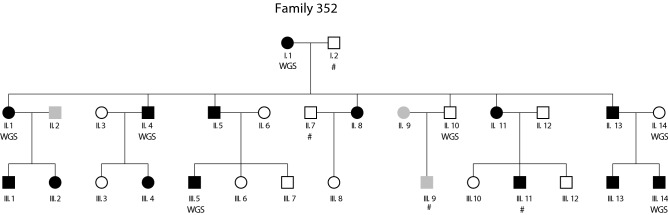
Pedigree of family 352. Black symbols represent individuals diagnosed with dyslexia, white symbols represent individuals without dyslexia, and gray symbols are individuals for which the phenotype was not known. #: individuals without DNA sample availability. *WGS* whole-genome sequence determined as part of this study

We studied a three-generation family of 30 members (referred to hereafter as family 352, Fig. 1), in which 14 relatives had a positive diagnosis of dyslexia. The inheritance pattern of the trait was consistent with the potential involvement of a single genetic locus, transmitted with an autosomal dominant mode of transmission. Specifically, individuals with dyslexia were observed in every generation, roughly half of the family members were affected, including similar numbers of males (*n* = 8) and females (*n* = 6), and there were three instances of male-to-male transmission (arguing strongly against X-linkage). This family, which has not been previously described in the literature, was recruited as part of a multidisciplinary research effort into different aspects of dyslexia (the Dutch Dyslexia Programme) (van der Leij and Maassen 2013), in which families were ascertained when at least two first degree relatives had a school history of reading problems (de Kovel et al. 2004; van der Leij and Maassen 2013). Informed consent was obtained from all participants, and the study was approved by the ethics committee (CWOM) of the University Medical Centre Nijmegen under CWOM-nr 9811-025.

### Phenotypic measures and diagnostic criteria

The family members were administered a battery of tests in single session, as briefly described below.

#### Word and non-word reading fluency

Word reading fluency was assessed using the One-Minute-Test (in Dutch, Een-Minute-Test or EMT), (Brus and Voeten 1972; Kuijpers et al. 2003), while non-word reading fluency was assessed using the Klepel test (van den Bos et al. 1994; Kuijpers et al. 2003). Participants were asked to correctly read as many items as possible within 1 min (word reading) or 2 min (non-word reading).

#### Verbal competence

Verbal competence was assessed as part of the Dutch version of the Wechsler Adult Intelligence Test (Uterwijk 2000), which tests the ability of the individual to express him/herself verbally. The participant is offered two words and is asked to describe as concisely as possible the similarities between them. Examples (in English) are *car-aeroplane* or *courage-cowardice*.

#### Diagnostic criteria

Dyslexia was defined following the criteria of the Dutch Dyslexia Programme, which were based on several reading-related quantitative measures (van der Leij and Maassen 2013). People were defined as affected if they (1) performed below the 10th percentile on a word reading test, or (2) scored below the 10th percentile on a non-word reading test, or (3) scored below the 25th normative percentile on both word and non-word reading tests, or (4) had a word or non-word reading score that was more than 60% points below their normalized score on a verbal competence test (discrepancy criterion). Following these criteria, 14 out of 27 tested family members were identified as having dyslexia. No sensory, developmental, or cognitive deficits that could confound the dyslexia diagnosis were detected among these individuals.

### Genotyping and linkage analysis

DNA was available for 26 individuals from family 352, including 13 with dyslexia, 11 without dyslexia, and two with unknown phenotype (i.e., all individuals except I.2, II.7, III.9, and III.11 in Fig. 1). These samples were genotyped using the Illumina Infinium OmniExpressExome-8 BeadChip (Illumina, Illumina Human OmniExpress-Exome, 2016a, b) by the genomics service company ‘Eurofins’ (Germany). Genotypes were called using Illumina GenomeStudio, using the “humanomniexpressexome-8v1-2_a.bpm” manifest file to map and annotate the SNPs to reference genome assembly build GRCh37/hg19.

A total of 7338 SNPs, with a minimum distance of 0.5 centimorgans (cM) between SNPs and an average heterozygosity of 49.7%, were selected for multipoint linkage analysis using LinkDataGen (Bahlo and Bromhead 2009), which also filtered out Mendelian inheritance errors and removed non-polymorphic SNPs within the family. Given the presence of three male-to-male transmissions in the family, which are not compatible with an X-linked inheritance pattern, chromosome X was not analyzed.

Multipoint parametric linkage analysis was performed using a dominant inheritance model, assuming a disorder allele frequency of 1% and penetrances of 5%, 99%, and 99% for wild-type, heterozygous, and homozygous mutation carriers, respectively. Given the uncertainty in defining a parametric model for this trait a priori*,* we also conducted non-parametric (NPL) multipoint linkage analysis, which does not rely on assumptions regarding monogenic inheritance, estimates of penetrance levels, and phenocopy rates (Fisher and DeFries 2002). Note that the NPL approach uses asymptotic large-sample assumptions to compute test statistics and approximate p values, so this method may not be optimal for single-pedigree analysis (McPeek 1999; Lange and Lange 2004). The NPL tests only for an increase in Identity-by-Descent allele sharing in affected individuals, without specifying a parametric model, but the lack of specification is accompanied by a loss of power.

Multipoint parametric linkage analyses were performed using the MORGAN (v3.2) programs lm_bayes (parametric) (George et al. 2005) and lm_ibdtests (non-parametric) (Basu et al. 2008), with 30,000 Monte Carlo iterations, and permutations for the non-parametric run. The NPL score was computed as the − log_10_(permuted *p* value).

We performed sensitivity analyses to confirm that the results were robust to different analytical options used within the parametric linkage analyses: the specific SNP-map selected (minimum distance between markers 0.3 cM or 0.5 cM) and the level of penetrances (0.05, 0.90, 0.90; 0.05, 0.95, 0.95).

#### Estimation of genome-wide significant linkage threshold and maximal possible LOD score

To derive an empirically determined threshold for genome-wide significant linkage, we carried out simulations, using the same SNPs that were included in the real linkage analysis. Permutations were performed using gene-dropping simulations, as implemented in MORGAN (v3.2). 1000 replicates were simulated, and each replicate was analyzed with the same parametric model as the real data using lm_bayes. The significance for each LOD score was assessed by: (1) counting the number of replicates (*n*) in which the maximum LOD score exceeded the highest observed LOD score and (2) calculating the *p* value as (*n* + 1)/1001. The threshold for genome-wide significant linkage, taken to be the 49th highest LOD score of the 1000 replicates, was estimated as 3.44. We also estimated the maximal possible LOD score that could be obtained in this family assuming an etiologic variant that cosegregates perfectly with the trait. To do so, we simulated a set of 10 consecutive biallelic markers (1 cM apart, Minor Allele Frequency, MAF = 0.25) whose allelic status fully segregated with dyslexia status (i.e., inherited by all 13 affected individuals but none of the 13 unaffected/unknown individuals) and ran parametric linkage analyses in an identical manner to the real data. In this scenario, we obtained a maximum LOD score of 5.3, clearly in excess of the empirical threshold for genome-wide significant linkage.

#### Haplotype analysis

To give further insights into regions showing strongest linkage to the trait, haplotypes were generated from SNPs covering the genomic region that showed multipoint LOD > 2. Haplotypes were created in the haplotype analysis tool simwalk2snp (Lange and Lange 2004) and visualized using Haplopainter (Thiele and Nurnberg 2005).

### Whole-genome sequencing

Genomic DNA samples collected from 7 members (5 affected and 2 unaffected, see Fig. 1) of family 352 were used for WGS by the genomics research organization and service company ‘Novogene’ (Hong Kong) using Illumina’s HiSeq Xten technology (Illumina, Illumina X-ten, 2016). The sample selection was motivated by the individuals’ quantitative trait scores (i.e., taking those most severely affected or obviously unaffected), and it was constrained by cost. One unrelated unaffected individual (Fig. 1, II.14) was also selected for WGS to help with filtering out possible sequencing artifacts. Sequencing was done at 30 times average coverage depth with library insert size of 350 base pairs and reads of 150 base pairs long (paired-end).

### Structural variant calling

Several signals in WGS data (e.g., read-pairs, split-reads, and read-depth) can indicate the presence of structural variants (SVs) (Mohiyuddin et al. 2015). Genotyping intensity data from SNP arrays can also be used to detect CNVs. Hence, we screened for different types of SVs using all the available data.

#### SVs from WGS

WGS data were used to call SVs, including deletions, duplications, and inversions. SVs were identified using three variant calling programs: CNVnator (Abyzov et al. 2011), BreakDancer (Chen et al. 2009), and Lumpy (Layer et al. 2014). Overlapping calls from the three SV detectors were then combined within samples using MetaSV (Mohiyuddin et al. 2015), considering as high-confidence those SVs that were detected by at least two of the tools. The seven WGS samples had on average 311,500 SV calls (range 310,900–311,900), of which on average 1915 (range 1855–1949) had been called by at least two different callers. SV calls across all samples were combined using the R package intanSV (Jia et al. 2020) by merging the calls that had been made in at least two samples with a reciprocal coordinate overlap larger than 10%. There were a total of 26,799 overlapped calls, 22,954 deletions, 3105 duplications, and 740 inversions.

The SV calls were compared against the Database of Genomic Variants (DGV, release July 2015; downloaded from UCSC genome browser hg19, February 2016) to annotate them as rare and common. Those that overlapped by > 50% of their lengths with five or fewer CNV events in the DGV were considered rare, while if they overlapped by > 50% of their lengths with more than five CNV events in the DGV, they were considered common. Those that did not overlap with any CNV in the DGV were classed as novel.

We excluded SVs as being potentially causative for the phenotype when they were present in the unaffected individual II.14 (married into family 352), or when they were common variants (as defined above) in the DGV database. IGV (Thorvaldsdottir et al. 2013) was used to visually evaluate putative SVs.

#### SVs from SNP microarrays

PennCNV (Wang et al. 2007) was used to detect CNVs from the signal intensity data. This program uses the normalized intensity data (Log R Ratio, and B Allele Frequencies) for SNP and CNV probes to identify putative CNVs using an HMM. For this analysis, we used default HMM parameters, as well as the PFB (Population Frequency of B allele) and GC files provided with the program (hhall.hg18.pfb, hhall.hg18.gcmodel), since our sample was too small to directly estimate these parameters from it. We used the joint calling option to take advantage of the family structure. Since only a trio-structure can be specified, a separate trio was defined for each non-founder sample. As a result, there were 1947 CNV calls, and each sample had on average 92.7 calls (range 31–211). CNV call coordinates were then lifted to hg19 genome reference to relate them to the rest of the results. We filtered out common CNVs (as defined for the DGV, see above) and CNVs that fell outside of the linked genomic regions (multipoint LOD > 1 or multipoint NPL > 1).

### WGS data processing

#### Alignment and pre-processing

Raw reads were cleaned by excluding adapter sequences, reads with low-quality bases for more than 50% of their lengths, and reads with unknown bases for more than 10% of their lengths. Clean reads comprised 97% of total reads, and were mapped onto the human reference genome (hg19) using the software Burrows–Wheeler Aligner (BWA) (Li and Durbin 2009). Bam files were sorted using SAMtools (Li et al. 2009) and PCR duplicate reads were marked using Picard (Picard 2016). The mean sequence read length was 150 base pairs, and approximately 97.5% of the genome was covered by at least a ten times sequence read depth.

Re-alignment around indels (insertion/deletions) and base quality control recalibration were performed using the Genome analysis toolkit software (GATK v3.4) (McKenna et al. 2010; DePristo et al. 2011).

#### Variant calling and annotation

Since more accurate variant calls can be achieved by including data from larger numbers of subjects simultaneously, we ran this process by pooling our data from family 352 together with 54 additional samples from different projects that had been sequenced with the identical protocols and in the same batches.

Genetic variants were called using the HaplotypeCaller (HC) tool of GATK (McKenna et al. 2010; DePristo et al. 2011). HC was run separately per sample using the ‘-ERC GVCF’ mode, and then merged together using the GenotypeGVCFs tool, as recommended by the GATK best practices (GATK v3.4). We performed Variant Quality Score Recalibration (VQSR) to exclude the low-quality variants (phred-scaled *Q*score < 30) and to flag the rest into the sensitivity tier they fell into (90, 99, 99.9, and 100). The variant calling of SNPs and indels identified on average 4,523,372 per sample (range: 4,455,342–4,581,631), for a total of 14,980,000 different variants across the 61 samples. These variants were then annotated using Annovar (Wang et al. 2010) and Variant Effect Predictor (VEP v37) (McLaren et al. 2010).

### Investigation of novel and rare variants

#### Imputation

To obtain genotype information on the family members that had not been sequenced, all novel and rare variants from WGS (i.e., having less than 1% frequency in the 1000 Genomes database, total = 306,597) were subjected to pedigree-based imputation. Gl_auto program from the MORGAN (v3.2) framework (Wijsman et al. 2006; Thompson 2011) was used to sample inheritance vectors from a Markov chain Monte Carlo analysis of the multilocus marker data (i.e., the 7338 SNPs that went into the linkage analyses). The inheritance vectors were used to impute genotype calls by GIGI (Cheung et al. 2013). The genotype probability threshold to call two alleles was set to 80%, and the threshold to call a single allele to 90%. The mean imputation rate was 51.58%. When the imputation resulted in haploid calls (i.e., only one allele imputed), we assumed that the unknown allele was the reference allele, since only rare variants were being imputed (The downstream analysis software to evaluate cosegregation of variants with dyslexia in the family required diploid genotypes).

#### Filtering of variants as potentially causative

7,805,631 variants had non-reference calls across the seven sequenced family members. The filtering strategy is shown in Fig. S1. First, we excluded variants if they were absent from all individuals with dyslexia, or if they were present in both unaffected members who had WGS data (2,707,476 variants kept). We then excluded common variants by filtering out any with reported MAF > 1% in 1000Genomes database (1000G), Exome Sequencing Project (ESP), and Exome Aggregation Consortium’s (ExAC) European populations (470,596 variants kept). Then, variants were excluded which fell outside genomic regions where linkage analysis found LODs or NPL scores exceeding 1, retaining 8528 variants. The use of a loose threshold for this variant filtering step allows for a thorough assessment that does not prematurely exclude potentially causative variants which imperfectly cosegregate with dyslexia in the family. As a final filter, we retained only those variants that were present in more than 60% of the individuals with dyslexia (2276 variants kept). Imputed genotypes were used for this filtering step. The filtered set of variants was then queried under different hypotheses considering coding and noncoding variation (Fig. S1):

(i) We identified two exonic variants, and excluded one annotated as a synonymous variant by both Annovar and VEP. (ii) Then, we identified 2274 noncoding variants, which we evaluated taking into account several scores (GWAVA, CADD, FIRE, see below) that aim to aid interpretation of noncoding regions of the genome. Note that precomputed scores are only available for SNVs and some indels, but not all noncoding indels could be evaluated by these scores. Unscored variants (559/2274) for which neither CADD, GWAVA, nor FIRE metrics were available were filtered out. Among the noncoding variants, we first considered those that were predicted to be likely pathogenic, based on GWAVA (Genome-Wide Annotation of Variants) (Ritchie et al. 2014) and CADD v1.6 (Combined Annotation Dependent Depletion) (Kircher et al. 2014; van der Velde et al. 2015) annotations. These two scores consider information on potential regulation of expression and evolutionary conservation, and each uses different algorithms (and assumptions) to evaluate the pathogenicity or functional importance of variants. The CADD metric measures deleteriousness by contrasting variants that survived natural selection (i.e., became fixed in the human lineage) with simulated mutations (Kircher et al. 2014), in such a way that variants with higher scores are likely to have been selected against (given their annotation pattern). The GWAVA score (Ritchie et al. 2014) uses similar sources of annotation, to discriminate between disease-causing (i.e., pathogenic) and control variants, and to apply this information to weight variants across the genome. We also considered noncoding variants based on the FIRE (Functional Inference of Regulators of Expression) score (Ioannidis et al. 2017), which is predictive of cis expression quantitative trait loci (eQTL), but does not rely on conservation information as GWAVA and CADD do. Thresholds were: GWAVA_unmatched_ > 0.5, CADD_phred_ > 15; FIRE > 0.6, the mean FIRE score value of autosomal cis-eQTL SNVs (Ioannidis et al. 2017). A full list of all 292 variants within the 7q21.11 linked region is presented (see “Results”), regardless of the availability of these scores.

We further assessed the putative functional impacts of the prioritized variants by checking available databases on regulatory regions of the genome [RegulomeDB (Boyle et al. 2012) and HaploReg (Ward and Kellis 2012)].

#### Association analysis

We performed a family-based test of allelic association (*M*_QLS_) using the *M*_QLS_-XM package (Thornton and McPeek 2007; Thornton et al. 2012), which does not assume a model. Rare variants (as defined above) located within genomic regions with multipoint LOD > 1 or NPL > 1 were included. Best-guess imputed genotypes were used for these analyses.

#### UK Biobank brain phenome scan

To characterize the phenotypic spectrum associated with variation at rs144517871, we conducted a phenome scan on 870 brain MRI phenotypes in the UK Biobank cohort, using PHESANT software (v0.13) (Millard et al. 2017). Analyses were restricted to participants of UK ancestry (UK Biobank specified variable) with brain imaging data. Individuals with high missingness (missing rate > 0.05), heterozygosity (PC corrected heterozygosity > 0.19), gender mismatch, and putative aneuploidies were excluded (Bycroft et al. 2018), as well as one from each pair of related individuals (i.e., with a kinship coefficient > 0.0442 as defined within the UK Biobank relatedness file). Genotype dosage for rs144517871 was converted into best-guess genotypes using PLINK v1.90b3w (Purcell and Chang 2020; Purcell et al. 2007). 33,441 individuals with non-missing genotypes (genotype AA = 32,689, CA = 749, CC = 3) were included in the analysis.

Linear regressions were fitted to test the association between genotypes of rs144517871 (AA = 0, CA = 1, CC = 2) and continuous brain measures outcomes. Analyses were adjusted for the following covariates: age at imaging (UKB field ID = f.21003_2_0), age^2^, sex (f.31_0_0), genotyping array (f.22000_0_0), 40 genetic principal components (f. 22009_0_1 to 22009_0_40), imaging assessment center (f.54_2_0), total gray matter volume (f. 25006_2_0), inverted signal-to-noise ratio in T1 (f.25734_2_0), inverted contrast-to-noise ratio in T1 (f. 25735_2_0), scanner lateral (X) brain position (f.25756_2_0), scanner transverse (Y) brain position (f.25757_2_0), scanner longitudinal (Z) brain position (f.25758_2_0), and scanner table position (f.25759_2_0). A conservative Bonferroni threshold was applied accounting for a total of 856 tests performed (*p* < 5.74 × 10^−5^). The UK Biobank data were obtained as part of research application 16066. The data collection for the UK Biobank has been described elsewhere (Sudlow et al. 2015). Informed consent was obtained by the UK Biobank for all participants.

#### Developmental expression pattern

The spatio-temporal expression patterns of *SEMA3C* during human development were characterized by querying the BrainSpan database (Miller et al. 2014).

### Luciferase reporter assay

An 1814 bp region of the *SEMA3C* promoter (from − 27 bp of the transcription start site to + 1244 bp of the first intron) containing the major allele of rs144517871 (A) was amplified from genomic DNA using the following primers: 5′ggtacccacccagcaagttgctcactcc3′ and 5′agatctgagacaggtgtcactgcttc3′. This construct was subcloned upstream of the luciferase reporter gene in the promoterless firefly luciferase vector pGL4.23 (Promega) between KpnI and BglII restriction sites. The rare rs144517871-C variant construct was generated by site-directed mutagenesis using the Quick-Change Site-Directed Mutagenesis kit (Stratagene) following the manufacturer’s protocol with the following primers: 5′ttgtgttggtaattttcaaaagcaacagtttcttcctgcttccac3′ and 5′ gtggaagcaggaagaaactgttgcttttgaaaattaccaacacaa3′. All constructs were verified by Sanger sequencing.

Luciferase assays were performed as described previously (Estruch et al. 2016). In brief, HEK293T and HeLa cells were seeded in clear-bottomed white 96-well plates and transfected in triplicate with equimolar concentrations of reporter constructs [5 ng of control reporter construct (pGL4.23) or 7.15 ng *SEMA3C* promoter-reporter construct (common: rs144517871-A or rare: rs144517871-C)], together with 4.96 ng of pRL-TK *Renilla* luciferase normalization control (Promega). After 48 h, luciferase activity was measured in a TECAN F200PRO microplate reader using the Dual-Luciferase Reporter Assay System (Promega). Relative luciferase expression was normalized to the control and results were presented as relative response ratios compared with the control reporter construct. Experiments were performed in triplicate and were repeated independently.

For each experiment, conditions were statistically compared: first, the minimal-promoter control vs the two allelic conditions, and next the two allelic conditions (common rs144517871-A and rare rs144517871-C) using an independent sample *t* test and a non-parametric Mann–Whitney–Wilcoxon rank test in R. We cannot verify the assumption of normality for the *t* test in these small numbers, but note that *t* tests are in principle robust in small samples if the assumptions are met. The non-parametric test provides an exact *p* value and is appropriate for relatively small numbers of observations in a given comparison, while the *t* test can have greater power when its assumptions are met. Results for all performed tests are reported.

## Results

We studied a large three-generation family (Fig. 1), in which almost half of the individuals were affected with dyslexia. The inheritance pattern was consistent with autosomal dominant Mendelian transmission, with affected members in every generation, 14 cases of dyslexia among the thirty family members, and three instances of male-to-male transmission. Simulations confirmed that for a family with this structure, the number of informative meioses is more than sufficient to detect genome-wide significant linkage, in the ideal case of a risk allele that perfectly cosegregates with the trait (see “Materials and methods”).

### Linked locus in chromosome 7q21

**Fig. 2:**
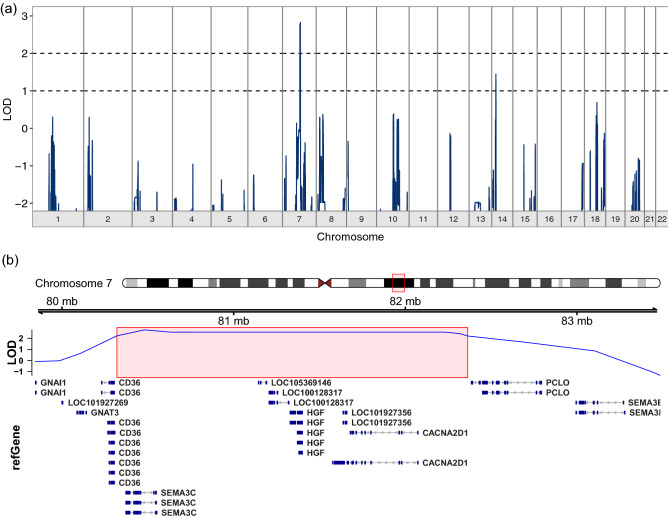
**a** Parametric multipoint linkage analysis across the genome. Chromosomes are represented along the *X* axis in numerical ascending order from left to right, and from their p to q arms. The *Y* axis shows the multipoint parametric linkage LOD score. **b** Genes under the highest linkage peak on chromosome 7q21.11, spanning physical positions 80–83 Mb on reference genome assembly build GRCh37/hg19. The red rectangle highlights regions showing multipoint LOD scores above 1.8 (LOD_max_ − 1). RefGene gene transcripts within the region are shown on the lower track

**Fig. 3:**
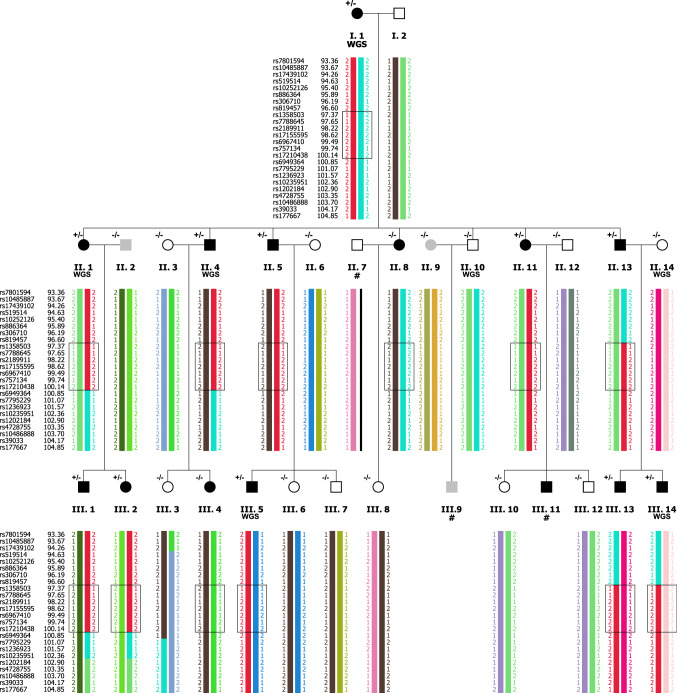
Haplotype visualization of the linked genomic region on chromosome 7q21.11. Individuals without a DNA sample are indicated by the # symbol. The putative ‘risk haplotype’ is shown in red. Upper and lower boundaries of the critical recombinations are marked within a box for all members with dyslexia (rs819457–rs6949364). Individuals II.8 and III.4 appear to be phenocopies (i.e., they have a diagnosis for dyslexia, but do not carry the risk haplotype in 7q21). Carrier status for rare minor allele of rs144517871 (confirmed via Sanger sequencing) is indicated above each individual, with −/− denoting wild type; +/− denoting heterozygous carrier

Multipoint linkage analysis using the SNP-chip data that we obtained from the available relatives did not detect a marker or haplotype that perfectly cosegregated with dyslexia, arguing against there being a simple Mendelian explanation for all the cases observed in the family. Indeed, no locus exceeded the genome-wide significance threshold for linkage (LOD of 3.44). The highest observed parametric LOD score was 2.83 on 7q21.11 (approximate 95% CI (Conneally et al. 1985) LOD_max_ − 1: chr7:80197286–83403157, hg19, see Fig. 2), in a region that encompasses several genes (*CD36, SEMA3C, LOC100128317, HGF, CACNA2D1, LOC101927356, PCLO*). Sensitivity analyses show that this is a robust signal across analysis options (Fig. S2). Analyses of marker haplotypes for 7q21.11 showed that this linked region was shared among 11 of the 13 affected family members, and none of the 13 unaffected/unknown members, for which DNA was available (Fig. 3). All chromosomal regions showing LOD or NPL scores exceeding 1 are shown in Table S1.

### No structural or coding variants cosegregate with dyslexia in the family

Based on analyses of SNP-chip and WGS data, there were no high-confidence SV or CNV calls that cosegregated with dyslexia or fell within regions defined by NPL or LOD > 1 (see Table S2 for a summary of low confidence calls within the suggestively linked regions).

Filtering of WGS data on MAF of the variants in public databases (1000G, ESP, and ExAC), and exclusion of synonymous variants yielded only one coding variant (chr13:111082914-A-T rs201716258, Table S3) located within the 13q34 NPL > 1 region. This missense variant (ENST00000360467.5:p.H203L) is within the *COL4A2* gene and is predicted to be benign/tolerated across prediction tools (Table S3). The number of variant carriers among the sequenced family members was: 4/5 affected and 0/2 unaffected, and in the imputed family members: 8/10 affected and 1/3 unaffected, which yields an *M*_QLS_ association p value of 0.11 (Table S3). *COL4A2* encodes a subunit of type IV collagen, a major structural component of basement membranes.

### Noncoding variants within the 7q21 linked locus

Table S4 shows all variants within the main linked region in chromosome 7q21.11 (292 variants).

**Fig. 4:**
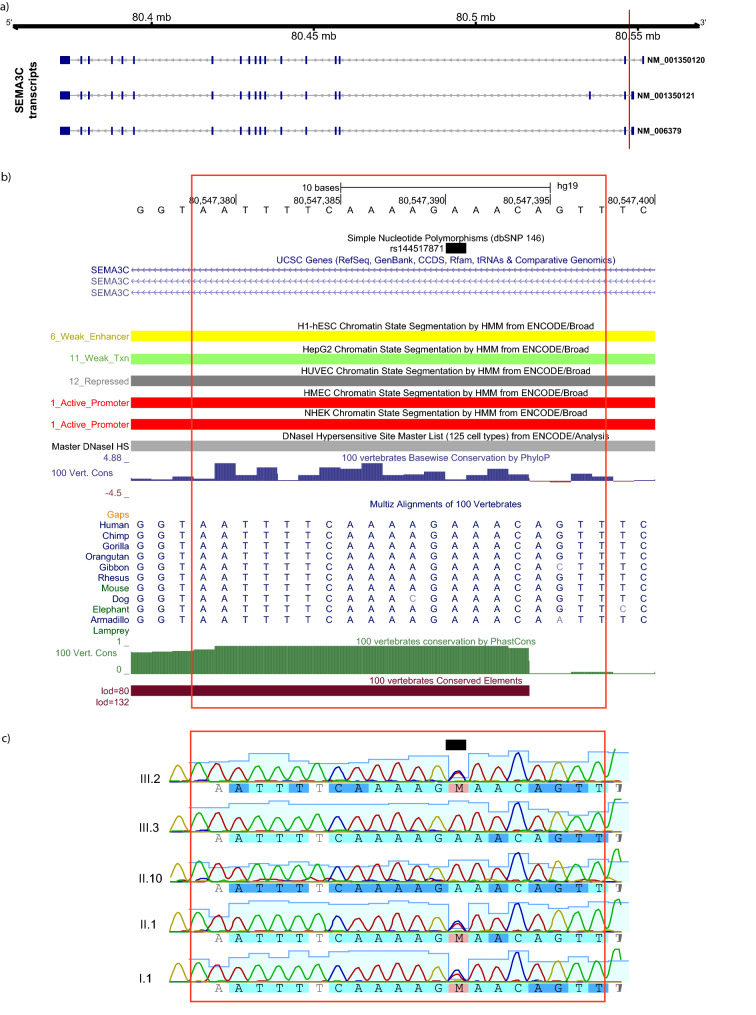
**a** Chromosome 7 genome axis and *SEMA3C* gene transcripts. The red bar indicates the genomic region where rs144517871 and rs143835534 are located. **b** Detailed annotation of the genomic region around rs144517871 (black square) using the UCSC Genome Browser (hg19). Tracks are included for ENCODE digital DNaseI HS hypersensitivity clusters, ENCODE/Broad chromatin state segmentation by Hidden Markov Model (HMM) in several cell lines, as well as 100 Vertebrate consevation scores (PhyloP, PhansCons, Conserved elements) and sequence alignment (Multiz Alignments of 100 Vertebrates). **c** Sanger sequencing chromatograms of rs144517871 for representative wild-type (A/A; individuals III.3, II.10) and risk-allele carrier (A/C; individuals III.2, II.1, I.1) family members

**Fig. 5:**
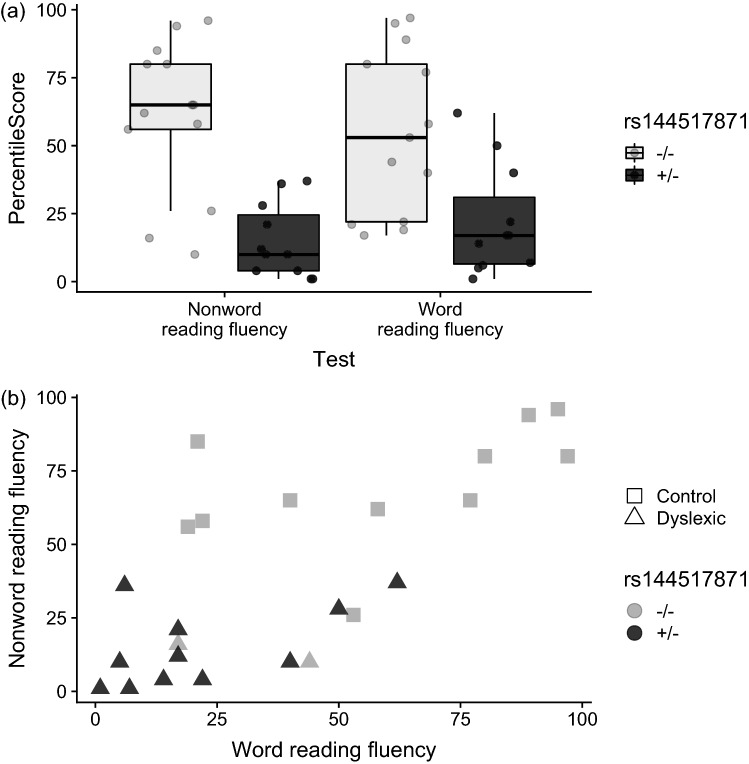
Reading scores for carriers (black, *n* = 11) and non-carriers (gray, *n* = 13) of the validated rs144517871 risk allele in family 352. **a** Percentile scores on word and non-word reading. **b** Relationship of the percentiles of the word reading and non-word reading tests. Triangles indicate individuals with a dyslexia diagnosis

There were 18 noncoding or synonymous variants within putatively linked genomic regions that were likely to be pathogenic based on aggregated annotation scores (CADD or GWAVA) (Table 1). Two of these variants (rs144517871 and rs143835534) fell within the most strongly linked region on chromosome 7q21.11. The two SNVs are 184 bp apart, and in perfect linkage disequilibrium (LD) (*r*^2^ = 1; 1000G eur RegulomeDB v3, Fig. S3) (Boyle et al. 2012), located within the first intron of the *SEMA3C* gene (Fig. 4a). The frequencies of both of the observed variants were 0.0031 in the 1000G overall population and slightly higher (0.0099) in the European subsample, as well as in a representative Dutch population (rs144517871 = 0.010 and rs143835534 = 0.009) (Genome of the Netherlands, http://www.nlgenome.nl/search/) (Francioli et al. 2014). The imputed genotypes predicted that both would cosegregate with dyslexia within the family, except for two putative phenocopies: II.8 and III.4 (i.e., matching the inheritance of the risk haplotype, Fig. 3). We further validated this finding in all the available DNA samples by Sanger sequencing of PCR products spanning rs144517871 (Fig. 4c). Each SNV yielded an *M*_QLS_ association p value of 0.02. The relationships of rs144517871 with word and non-word reading fluency (Fig. 5) showed that risk-allele carriers performed more poorly overall than non-carriers on both measures, although this is confirmatory and not independent evidence, as these measures were used to define cases and controls in the family (“Materials and methods”).

**Table 1:** Rare, noncoding variants mapping within genomic regions and predicted by CADD/GWAVA to be pathogenic

Chr	Start	Ref	Alt	cytoBand	snp138	Gene	Func	MAF_1000G_	CADD	GWAVA	FIRE	M_QLS_	Affected	Unaffected	Imp Rate	Linked region
1	68296119	T	C	1p31.3	rs111907541	*GNG12*	intronic	0.006	5.78	0.64	0.41	0.07	0.67	0.00	0.30	NPL > 1
1	68300052	G	A	1p31.3	rs111472994	*GNG12-AS1*	ncRNA intronic	0.006	15.51	0.84	0.29	0.07	0.67	0.00	0.30	NPL > 1
1	68627652	C	G	1p31.3		*GNG12-AS1*	ncRNA intronic		15.67	–	0.55	0.02	1.00	0.50	0.30	NPL > 1
1	68627723	T	C	1p31.3		*GNG12-AS1*	ncRNA intronic		15.92	–	0.56	1.00	1.00	–	0.11	NPL > 1
1	68628015	T	G	1p31.3		*GNG12-AS1*	ncRNA intronic		16.84	–	0.51	0.07	0.67	0.00	0.30	NPL > 1
1	68628019	G	A	1p31.3		*GNG12-AS1*	ncRNA intronic		16.78	–	0.52	0.07	0.67	0.00	0.30	NPL > 1
1	68628027	G	A	1p31.3		*GNG12-AS1*	ncRNA intronic		15.74	–	0.55	0.07	0.67	0.00	0.30	NPL > 1
1	68628031	A	T	1p31.3		*GNG12-AS1*	ncRNA intronic		16.05	–	0.53	0.07	0.67	0.00	0.30	NPL > 1
1	68628752	T	A	1p31.3	rs17842969	*GNG12-AS1*	ncRNA intronic		15.63	–	0.55	–	1.00	1.00	0.19	NPL > 1
1	68628754	T	C	1p31.3	rs17090698	*GNG12-AS1*	ncRNA intronic		16.09	–	0.56	–	1.00	1.00	0.19	NPL > 1
1	70507620	G	A	1p31.1	rs189591558	*LRRC7*	intronic	0.001	6.48	0.51	0.48	0.23	0.83	0.50	0.30	NPL > 1
2	5865630	G	A	2p25.2	rs145390307	*SOX11, LINC01105*	intergenic	0.005	0.48	0.65	0.63	0.23	0.70	0.00	0.48	NPL > 1
2	6485809	T	C	2p25.2	rs146498852	*LOC400940, LINC01247*	intergenic	0.007	21.80	–	0.36	0.23	0.70	0.00	0.48	NPL > 1
2	6776265	T	C	2p25.2	rs142428415	*LINC01246, MIR7515HG*	intergenic	0.007	2.97	0.63	0.42	0.23	0.70	0.00	0.48	NPL > 1
2	7150786	A	G	2p25.1	rs141684587	*RNF144A*	intronic	0.007	15.32	0.21	0.40	0.23	0.70	0.00	0.48	NPL > 1
3	9079126	–	A	3p25.3	rs35966925	*SRGAP3*	intronic		15.41	–	–	0.19	0.80	0.67	0.48	NPL > 1
4	98275548	T	C	4q22.3		*PDHA2, STPG2-AS1*	intergenic		15.17	–	0.08	0.51	0.67	0.00	0.41	NPL > 1
7	80547391	A	C	7q21.11	rs144517871	*SEMA3C*	intronic	0.0099	17.96	0.82	0.38	0.02	0.90	0.00	0.52	LOD > 1
7	80547575	T	C	7q21.11	rs143835534	*SEMA3C*	intronic	0.0099	3.10	0.74	0.45	0.02	0.90	0.00	0.52	LOD > 1

Genomic regions of interest are defined in Table S1. Min: minor allele. MAF: minor allele frequency in the 1000G (eur) population. CADD: deleteriousness score. GWAVA: functional impact score. M_QLS_: association *p* value. ImpRate: imputation rate across the members of family 352 without sequencing data. Affected, unaffected/unknown: frequency of variant carriers of each type in family 352. The frequency of carriers is given for the imputed genotypes within each group

*SEMA3C* is most highly expressed during early brain development in cortical regions, with expression levels decreasing after birth (Miller et al. 2014). We investigated potential associations of rs144517871 allelic variation with aspects of brain structure and function in adults from the general population, by performing a phenome-wide scan of 856 magnetic resonance imaging (MRI) phenotypes in the UK Biobank (*N* = 33,441), as implemented in PHESANT (Millard et al. 2017). No associations were found that were significant after multiple comparison correction; the strongest evidence was found for the superior part of the precentral sulcus in the left hemisphere (“Area of S-precentral-sup-part (left hemisphere)”), with the minor allele of rs144517871 (C) showing association with a decrease in the cortical surface area of this region (beta = − 0.018, nominal *p* = 4.9 × 10^–4^).

As a complementary approach to assess putatively functional noncoding variants, we checked variants with a high FIRE score, which predicts cis-eQTL function without making assumptions about pathogenicity. Thirty-four noncoding variants within regions LOD > 1 or NPL > 1 were likely to regulate gene expression, with a FIRE score larger than 0.6 (Table S5). Of these, none fell within the main linked locus (7q21.11), and none was associated with dyslexia status (*M*_QLS_ *p* > 0.05, Table S5).

### Rs144517871 modulates gene expression in a reporter assay using human cell lines

To determine if allelic variation of rs144517871 has potential to affect the transcriptional regulatory activity of *SEMA3C*, we performed a luciferase reporter assay in different cell lines. HeLa or HEK cells were transfected with a reporter construct in which expression of the luciferase gene was driven by a region of the *SEMA3C* promoter containing either the common (A) or rare (C) allele of rs144517871, or a control reporter vector in which the luciferase gene was placed upstream of a minimal promoter.

**Fig. 6:**
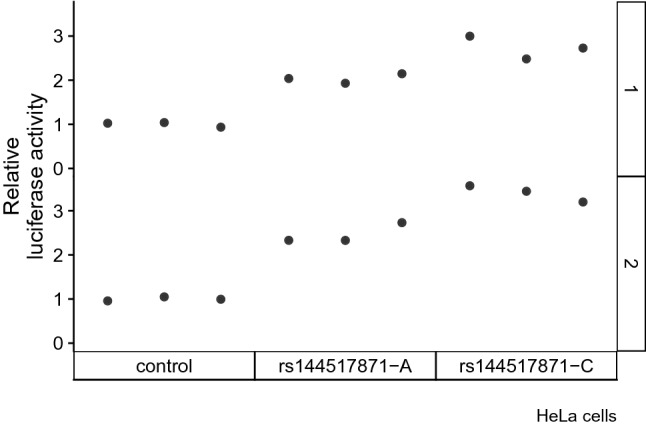
Luciferase reporter assays HeLa cells. Luciferase reporter vector containing a minimal promoter (control) or *SEMA3C* promoter containing either the common allele (rs144517871-A) or the rare allele (rs144517871-C) were transfected into HeLa cells. Luciferase expression was measured, and data are shown as relative ratio to the control construct. The two panels (1 and 2) indicate independent experimental replicates

The promoterless condition showed lower relative luciferase activity compared to the two allele conditions (A and C), indicating that the *SEMA3C* promoter region is sufficient to increase luciferase gene expression (Tables S6 and S7; HeLa *t* test *p* values < 0.001 and Mann–Whitney–Wilcoxon *U* test *p* values = 0.02; HEK *t* test *p* values = 0.0003 and 0.07 and Mann–Whitney–Wilcoxon *U* test *p* values = 0.02 and 0.17). We observed an increase of activity relative to the common variant when the rare rs144517871-C allele was present in HeLa (Fig. 6, Table S6 and S7) and HEK cell lines (Fig. S4, Table S6 and S7) (all *t* test *p* values < 0.05; all Mann–Whitney–Wilcoxon *U* test *p* values = 0.1).

## Discussion

In the present study, we adopted a strategy of combining linkage analysis with WGS to identify genetic variants that may contribute to dyslexia in a large three-generation family in which around half of the relatives are affected.

Parametric multipoint linkage analysis under a dominant mode of inheritance in family 352 identified the strongest evidence for a region on chromosome 7q21.11 (LOD = 2.83). With the exception of two affected members that were putative phenocopies, the identified risk haplotype cosegregated perfectly with dyslexia status in the family: it was found in a heterozygous state in 11 members with dyslexia while being absent from 11 members without dyslexia and two of unknown phenotype. The linked region encompassed several genes (*CD36, SEMA3C, LOC100128317, HGF, CACNA2D1, LOC101927356, PCLO*). Within this region, there were two rare (MAF < 0.01), noncoding SNVs (rs144517871 and rs143835534) predicted to have functional effects, located in the first intron of the gene *SEMA3C*, and in high LD with each other. These variants cosegregated perfectly with the risk haplotype. In silico characterization showed that rs144517871 affects an evolutionarily constrained regulatory region in the first intron of *SEMA3C*, located between exons that are part of the 5′ untranslated region of the transcript. This SNP can act also as a cis-eQTL for *SEMA3C* (GTEx Consortium 2015). To functionally test whether such an effect on gene regulation exists, we performed luciferase reporter gene assays; in this way, we showed that this region increases gene expression and that its regulatory activity is modulated by the allelic status of rs144517871, with the rare variant resulting in significant increases in expression levels. These putative effects on expression are subtle and should be validated further using independent in vitro and in vivo systems. Nevertheless, since dyslexia is a complex and relatively subtle phenotype, genetic contributions to it need not have severe consequences at the molecular/cellular level. A regulatory mechanism that affects the abundance of mRNA/protein, rather than disrupting the function of the encoded protein itself, is biologically plausible. These results are in line with the previous studies in which DNA variations potentially associated with dyslexia were shown to affect the expression of proximal genes (Hannula-Jouppi et al. 2005; Dennis et al. 2009)*.* We performed a phenome scan of MRI-based brain measures using available UK Biobank data from healthy adults, but did not observe significant associations that survived correction for the number of tested phenotypes.

The rs144517871 variant is located within a predicted regulatory region according to the EnsemblRegulatory Build (Zerbino et al. 2015) (Fig. 4b), and has high scores for potential functional impact, CADD = 17.96 and GWAVA = 0.82. CADD and GWAVA aggregate annotations on potential regulation of expression and evolutionary conservation to evaluate the pathogenicity or functional importance of variants (with CADD > 15 considered to be deleterious, and GWAVA > 0.5 pathogenic). The FIRE score for rs144517871 was 0.45. Higher FIRE scores (range 0–1) indicate that SNVs are more likely to alter the expression levels of nearby genes (Ioannidis et al. 2017). According to Haploreg (Ritchie et al. 2014), rs144517871 has promoter and enhancer histone marks in multiple tissues including brain (17 and 4 tissues respectively; see Fig. S5). Importantly, the allelic variation at this site is predicted to alter several regulatory motifs [Haploreg: Pou2f2, Pou5f1, ZEB1, Table S8 (Ward and Kellis 2012); Regulome: Pou5f1 (Boyle et al. 2012)] and the site is well conserved across mammals, with a GERP conservation score of 4.73. The other variant in perfect LD within the *SEMA3C* intron, rs143835534, has lower functional prediction scores: CADD = 6.57 (i.e., not deleterious) and GWAVA = 0.74 (i.e., predicted pathogenic). This variant shares some of the histone marks with rs144517871, and is predicted to alter some regulatory motifs, but does not show evolutionary conservation (GERP = − 1.28) (Fig. S3 and S5). We checked whether either of the two variants (or any other variant in high linkage disequilibrium, Fig. S5) was predicted to act as an expression quantitative trait locus (eQTL) in public databases [Braineac and Genotype Tissue Expression, GTEx, V7 Ramasamy et al. 2014; GTEx Consortium 2015)]. Both variants predicted the expression of *SEMA3C* in blood (uncorrected *p* value 0.0021).

The interpretation of functional annotation of variants outside protein-coding regions is complex. In this study, we used CADD and GWAVA summary scores (Kircher et al. 2014; Ritchie et al. 2014) that integrate information from different sources at the DNA and protein levels and genomic properties to rank variants according to expected deleteriousness (CADD) or pathogenicity (GWAVA). We took advantage of these metrics to rank the variants, as a proxy for affecting gene function, but it should be noted that since dyslexia occurs in otherwise healthy individuals, and is unlikely to reduce the chance of having children, we cannot assume that causal variants have been subjected to negative selection. Nevertheless, a variant that increases the risk of a trait like dyslexia in the dominant form (i.e., when heterozygous), could lead to a more severe disorder in recessive form (i.e., when in a homozygous state or as a compound heterozygote with another deleterious variant of the same gene). Variants may also have pleiotropic effects, impacting multiple traits with differential severity. Both tools predicted that rs144517871 has functional effects, which we were able to validate experimentally by performing luciferase reporter assays in human cell lines.

*SEMA3C* encodes a class III semaphorin. These secreted proteins bind to plexin and play an important role in the regulation of developmental processes, including providing guidance cues to migrating cortical neurons (Chen et al. 2008; Van Battum et al. 2015). In the embryonic mouse brain, *Sema3c* is transcriptionally repressed by Bcl11a in radially migrating neurons (Wiegreffe et al. 2015). Wiegreffe et al (2015) found that homozygous mutant *Bcl11a* mice presented defects in neuronal morphology and neuronal migration, and that the mutant phenotype was rescued when knocking down *Sema3c*. Of note, a de novo microdeletion in the dyslexia susceptibility locus 3 (DYX3) on chromosome 2p spanning only the *BCL11A* gene was reported in a proband with severe speech sound disorder (Peter et al. 2014). Furthermore, heterozygous de novo mutations in *BCL11A* are associated with an intellectual disability syndrome involving delayed speech and language, and haploinsufficiency for *Bcl11a* in mice also resulted in postnatal upregulation of class III semaphorins in the cortex (*Sema3d*) and hippocampus (*Sema3e*) (Dias et al. 2016). Due to its roles in brain development, *SEMA3C* represents a convincing candidate gene for susceptibility to dyslexia, a cognitive trait which has been associated with changes in cerebral cortical architecture (Giraud and Ramus 2013).

A previous study identified linkage with dyslexia spanning the same location on 7q21.11 (multipoint LOD = 3.08, microsatellite marker at linkage peak = D7S660) in families diagnosed with Rolandic epilepsy (Strug et al. 2012). *SEMA3C* was the closest gene to the peak of linkage, and the authors screened the protein-coding and promoter regions of the *SEMA3C* gene for mutations in one of the families that contributed most to their linkage LOD score in this study. However, they were unable to identify mutations that cosegregated with dyslexia, and suggested that either intronic regions or other genes could be responsible for the signal. In light of our data from the present study, it could be worth further investigating the families studied by Strug et al. (2012) with a focus on potential regulatory variation affecting *SEMA3C*, to assess whether such variation is relevant to dyslexia in the context of rolandic epilepsy. We could not address this issue in the present study, as family 352 was recruited specifically through a diagnosis of dyslexia.

We further examined if any rare coding variants could be contributing to dyslexia in this family. A missense SNV in *COL4A2*, predicted to be tolerated, was the only coding non-synonymous mutation within regions showing an NPL > 1. This gene has been associated with “Brain small vessel disease 2”(OMIM 614483), an autosomal dominant cerebrovascular disorder characterized by variable neurologic impairment resulting from the disturbed vascular supply that leads to cerebral degeneration (Meuwissen et al. 2015). Although a common SNP in *COL4A2* (rs9521789) was associated with comorbid reading disability and language impairment in an early genome-wide association study (Eicher et al. 2013), the SNP showed no association with reading-related quantitative traits in two other samples (Eicher et al. 2013; Carrion-Castillo et al. 2016).

Of note, the variants identified in the present study are not novel mutations, since they are present in the general population at low frequencies. Hence, these variants could be considered risk factors that are particularly penetrant in this family, possibly acting in coordination with other genetic and/or environmental effects. Further exploration of “gene × gene” and “gene × environment” interactions with respect to these variants in large population cohorts in future work may help elucidate such mechanistic aspects.

In summary, a combination of linkage analysis and WGS was used to search for rare DNA variants segregating with dyslexia in an extended Dutch family. This strategy has proven to be effective for finding causative variants involved in several monogenic and more complex traits (Rosenthal et al. 2013; Norton et al. 2013; Corominas et al. 2018). We identified a region on chromosome 7q21.11 linked to dyslexia, which was concordant with linkage findings with dyslexia reported in a previous study of rolandic epilepsy families (Strug et al. 2012). There were no rare or novel exonic variants within this region. Instead, we found that two relatively rare (MAF ~ 0.01) noncoding variants within the first intron of the gene *SEMA3C,* predicted to have a functional impact, were present in all but two family members with dyslexia, while being absent from all unaffected members. Histone marking, eQTL data, and experiments with luciferase reporter assays, provided evidence that these intronic variants could have a *cis*-regulatory effect on the expression levels of the gene. We thus propose *SEMA3C* as a candidate gene for dyslexia susceptibility.

## Supplementary Information

Below is the link to the electronic supplementary material.

Supplementary file1 (XLSX 151 kb)

Supplementary file2 (PDF 468 kb)

## Data Availability

Anonymized primary data are deposited at The Language Archive (TLA: https://archive.mpi.nl/), a public data archive hosted by the Max Planck Institute for Psycholinguistics. Data are stored at the TLA and accessible with persistent identifiers: https://hdl.handle.net/1839/2f897bc7-bc62-4b27-9fe8-8d02ec6c822c and https://hdl.handle.net/1839/c908dc8a-ec12-4767-b76d-1933c17be01c. Access can be granted upon request.

## Code availability

Custom codes for the analyses in this study are deposited at The Language Archive (TLA: https://archive.mpi.nl/), a public data archive hosted by the Max Planck Institute for Psycholinguistics. Scripts and output files are stored at the TLA and accessible with persistent identifiers: https://archive.mpi.nl/mpi/islandora/object/mpi%3A1839_fe2ba831_974d_4ec0_92f1_41b99c82591a (whole-genome sequencing—pre-processing and calling) and https://hdl.handle.net/1839/7ec56abe-a2d9-435d-bd6c-4f1960867b4e (linkage and NGS processed data). Access can be granted upon request.
