# Supplementary material for: Whole-genome sequencing identifies functional noncoding variation in SEMA3C that cosegregates with dyslexia in a multigenerational family
Source: Hum Genet. 2021 Jun 2;140(8):1183–200. doi: 10.1007/s00439-021-02289-w (PMC8263547; doi:10.1007/s00439-021-02289-w)
Supplement: Supplementary file 2 — Supplementary file2 (PDF 468 kb) [file 439_2021_2289_MOESM2_ESM.pdf]

Supplementary Figures

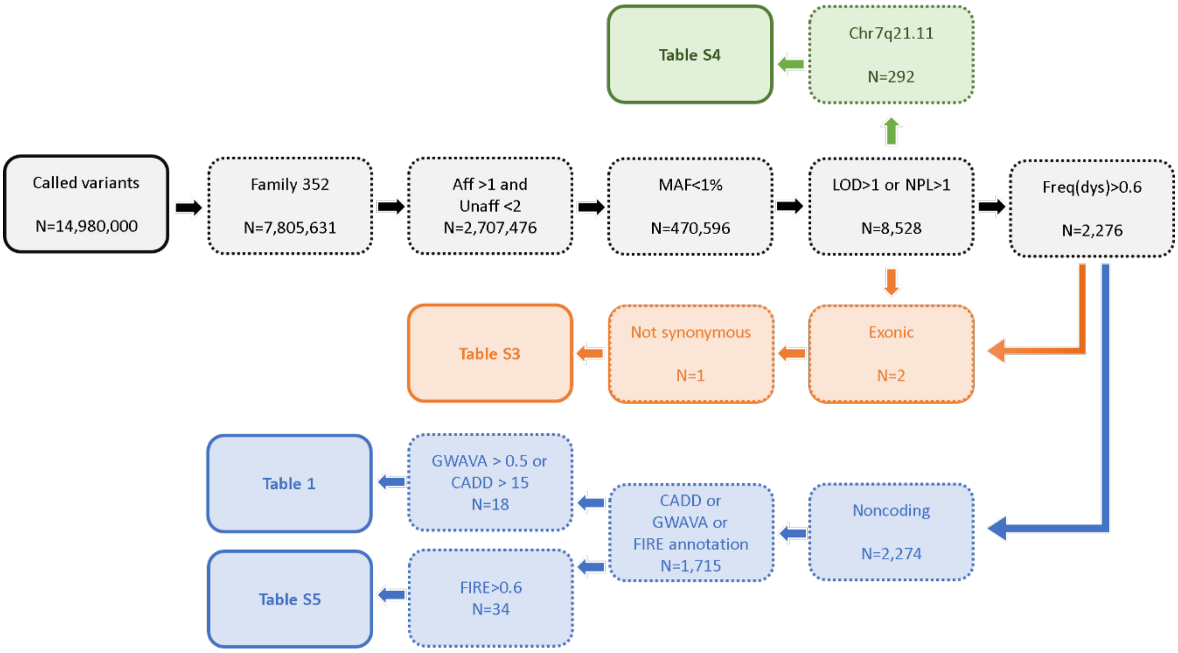

Fig S1.WGS filtering diagram. Dotted squares indicate the applied filters. Solid squares indicate the starting variant calls (black) and the final tables for the exonic (orange) and noncoding (blue) variants.

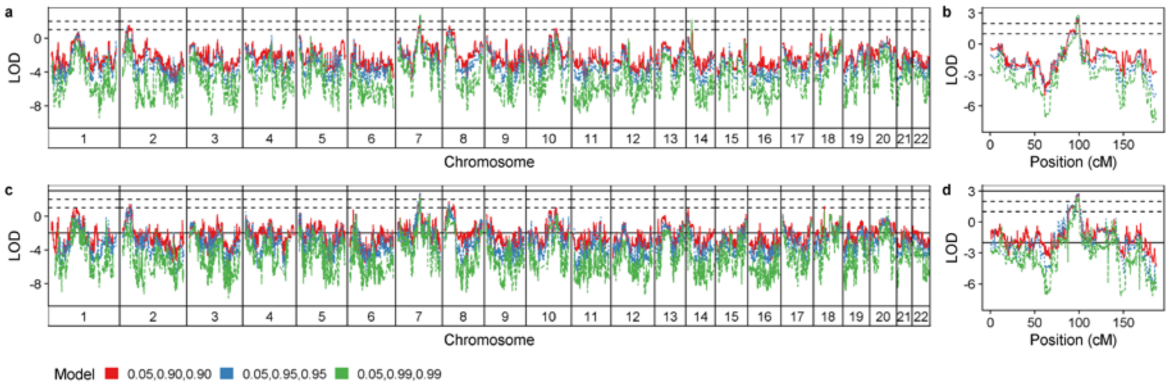

Fig S2. Sensitivity analysis of parametric dominant linkage. a) and **b)** map bin-size: 0.5 cM, **c)** and **d)** map bin-size: 0.3 cM. Colours indicate models with different penetrances for wild-type, heterozygous, and homozygous allele carriers: red (0.05,0.90,0.90), blue (0.05,0.95,0.95) and green (0.05,0.99,0.99). All autosomal chromosomes are shown in **a** and **c**, and chromosome 7 is shown in **b** and **d**.

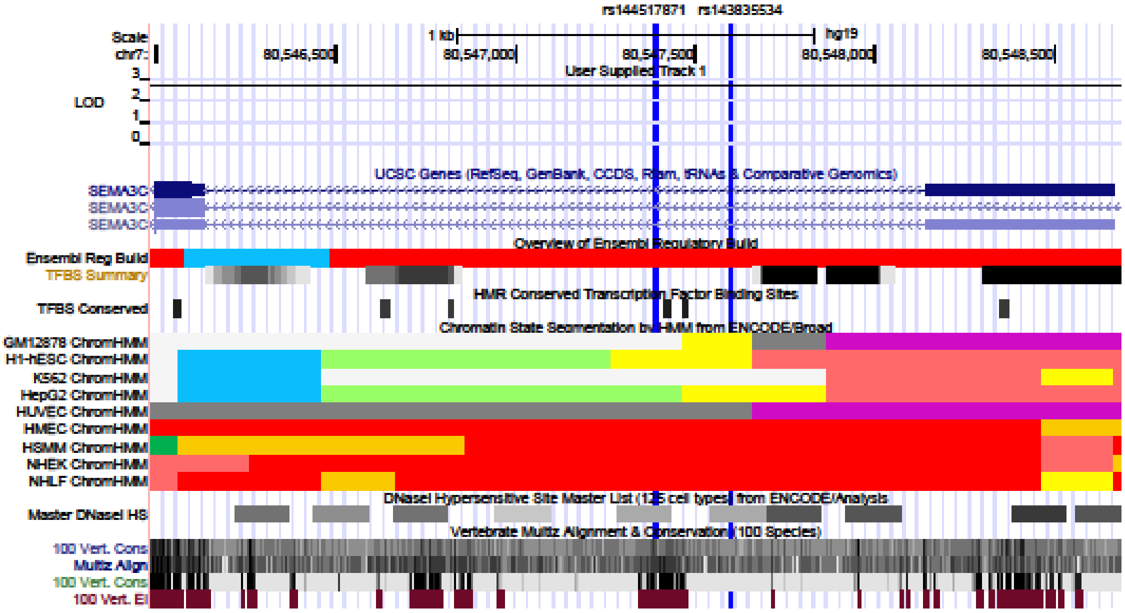

Fig S3. Detailed annotation of the genomic region first intron of *SEMA3C* using the UCSC Genome Browser (hg19). The variants rs144517871 and rs143835534 are marked in blue. Tracks for ENCODE digital DNaseI HS hypersensitivity clusters, transcription factor binding sites, ENCODE/Broad chromatin state segmentation by HMM in several cell lines, as well as 100 Vertebrate consevation scores (PhyloP, PhansCons, Conserved elements) and sequence alignment (Multiz Alignments of 100 Vertebrates) are included.

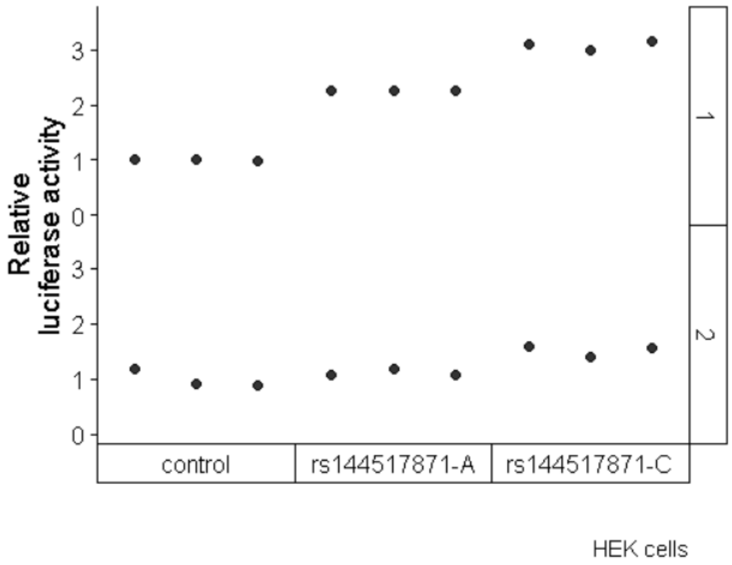

Fig S4. Luciferase reporter assays in HEK cells. Luciferase reporter vector containing a minimal promoter (control) or *SEMA3C* promoter containing either the common allele (rs144517871-A) or the rare allele (rs144517871-C) were transfected into HEK cells. Luciferase expression was measured and data are shown as relative ratio to the control construct. The two panels (1 and 2) indicate independent experimental replicates.

Query SNP: rs144517871 and variants with r^2 >= 0.2

pos (hg19)	pos (hg38)	LD (r^2)	LD (D')	variant	Ref	Alt	AFR freq	AMR freq	ASN freq	EUR freq	SiPhy cons	Promoter histone marks	Enhancer histone marks	DNAse	Proteins bound	eQTL tissues	Motifs changed	Drivers disrupted	GENCODE genes	dbSNP func annot
chr7:80387225	chr7:80757909	0.22	0.62	rs192757316	T	G	0.00	0.00	0.00	0.02							ERalpha-a,TLX1::NFIC		SEMA3C	intronic
chr7:80406219	chr7:80776903	0.4	0.75	rs142143424	T	C	0.00	0.00	0.00	0.01			BRST				Arnt,BHLHE40,XBP-1		SEMA3C	intronic
chr7:80452285	chr7:80822969	0.24	0.49	rs149488275	C	T	0.00	0.00	0.00	0.01			ESDR, BRST, GI, KID, LNG	MUS,MUS			Foxl1,Hoxa10,Hoxa9,NF-AT,Pou1f1,STAT		SEMA3C	intronic
chr7:80547391	chr7:80918075	1	1	rs144517871	A	C	0.00	0.00	0.00	0.01		ESDR, ESC, LNG, IPSC, FAT, STRM, BRST, BLD, MUS, SKIN, VAS, BRN, GI, KID, HRT, OVRY, BONE	ESC, IPSC, BLD, BRN, HRT, LNG, CRVX	BRN,MUS,MUS,BRST			GR,Pou2f2,Pou5f1,ZEB1		SEMA3C	intronic
chr7:80547575	chr7:80918259	1	1	rs143835534	T	C	0.00	0.00	0.00	0.01		ESC, ESDR, LNG, IPSC, FAT, STRM, BRST, BLD, MUS, SKIN, VAS, BRN, GI, KID, HRT, OVRY, CRVX, BONE	ESC, IPSC, HRT, LNG	ESDR,LNG,MUS,CRVX			GATA,GR,Pax-4		SEMA3C	intronic
chr7:80573237	chr7:80943921	0.25	1	rs190412691	T	C	0.00	0.00	0.01	0.00			LNG, BRST, BLD, SKIN						22kb 5' of SEMA3C	
chr7:80583080	chr7:80953764	0.5	1	rs182566272	G	C	0.00	0.00	0.00	0.01							Cdx2,Hoxa10,Hoxd10,Sox,VDR		31kb 5' of SEMA3C	
chr7:80607490	chr7:80978174	0.25	1	rs146727624	G	A	0.00	0.01	0.00	0.00							AP-1,CIZ,Ik-1		56kb 5' of SEMA3C	

Fig S5. Haploreg annotations of the noncoding variants in linkage disequilibrium (r2>=0.2) with rs144517871. Predicted chromatin state is shown per tissue. Effect on regulatory motifs is shown.
